# A Multimodal Approach to Lateral Canthotomy and Cantholysis Training for Emergency Medicine Trainees: A Simulation Training Package

**DOI:** 10.5070/M5.52351

**Published:** 2026-01-31

**Authors:** Haris Shoaib, Yunus K Hussain, Shiza Shoaib, Sulaiman Hussain, Haider A Chaudhary, Muhammad Subed Ali, Cara Jennings, Tara Smith

**Affiliations:** 1 Royal Bolton Hospital, Department of Trauma & Orthopaedics, Bolton, United Kingdom; 2 Guy’s and St Thomas’ NHS Foundation Trust, Department of Radiology, London, United Kingdom; 3 Guy’s Campus, King’s College Hospital, GKT School of Medical Education, London, United Kingdom; 4 Royal Blackburn Hospital, Emergency Department, Lancashire, United Kingdom; 5 Cumberland Infirmary, Department of Oral & Maxillofacial Surgery, Cumbria, United Kingdom; 6 Basildon & Thurrock University Hospital, Department of Clinical Education, Essex, United Kingdom; 7 King’s College Hospital, Emergency Department, London, United Kingdom

## Abstract

**Audience:**

This simulation is intended for emergency medicine residents.

**Background:**

Lateral canthotomy and cantholysis (LCC) is a sight-saving procedure for orbital compartment syndrome (OCS).^1^ Due to the rarity of OCS, emergency-medicine trainees often have limited exposure and low procedural confidence. In a questionnaire we found that trainees have low confidence levels in performing the procedure attributed to the low incidence of OCS and the scarcity of training opportunities. Existing literature describes LCC task trainers with creation of simulation models, but few provide a reproducible multimodal package adaptable for multiple training centres.2,3 Our innovation combines presentation, instructional video, gamified quiz, and hands-on practicer with low fidelity models. Unlike previous studies referencing the use of pre-made or cadaveric-based models, this design offers detailed guidance on model assembly using commonly available and low-cost materials.2,3 We designed and tested a multimodal training approach to optimize trainee confidence and competence in recognizing OSC and performing the LCC procedure. This aligns with recent calls in medical education for innovative, cost-effective simulation that maintains clear guidance and instructions while overcoming financial and/or logistical barriers.4,5

**Educational Objectives:**

By the end of this session, learners should be able to: 1) recognize the clinical features of OCS, 2) describe the indications and steps of performing LCC, 3) perform a lateral canthotomy and cantholysis procedure on a low-fidelity model, and 4) demonstrate improved confidence in recognizing and managing OCS.

**Educational Methods:**

The training uses a multimodal structure involving the following resources: 1) Instructor-led presentation on OCS and LCC with a step-by-step guide (Appendix A), 2) gamified quiz (Appendix B), 3) a procedural demonstration video, 4) a procedural handout containing a step-by-step guide (Appendix C), 5) a low-fidelity model of the orbit constructed from inexpensive materials, and 6) hands-on procedural practice with trainer feedback.

**Research Methods:**

Trainees’ confidence and perceived competence in performing the procedure were assessed using a 10-point Likert scale before and after the training, in addition to collection of qualitative feedback via free-text comments. Trainees also rated all educational components of the course using a 10- point Likert scale. Statistical significance was calculated using paired t-tests.

**Results:**

A total of thirty-four emergency medicine trainees participated in this multi-national training simulation package across three centers within the UK, completing pre- and post-intervention feedback. We observed a significant improvement in participants’ self-assessed confidence levels when comparing theoretical knowledge (5.0 ± 2.5 to 8.7 ± 1.7; p < 0.0001) and procedural competence (4.1 ± 2.8 to 8.9 ± 1.5; p < 0.0001). All educational components of the training package were rated highly, with mean scores ranging between 8.7 and 10 (measured on a 10-point Likert scale). All trainees involved supported the idea of annual delivery of the training package to emergency medicine trainees. Qualitative feedback further supported the value of practical simulation.

**Discussion:**

Given the severe clinical consequences of OCS, there is a need for diagnostic and procedural competence. This training package demonstrated significant improvement in trainees’ confidence and competence for a rare but critical emergency department procedure. The low-fidelity model and simulation package is reproducible, cost-effective, and scalable across training centres.

**Topics:**

Simulation, emergency medicine, orbital compartment syndrome, lateral canthotomy, cantholysis, procedural skills, low-fidelity model.

## USER GUIDE

**Table:** 

List of Resources:	
Abstract	1
User Guide	3
Appendix A: PowerPoint Presentation	9
Appendix B: Quiz Questions and Answers	10
Appendix C: Lateral Canthotomy and Cantholysis Procedural Handout (Step-by-Step Guide)	14
Appendix D: Pre-Intervention Feedback	16
Appendix E: Post-Intervention Feedback	17

**Learner Audience:**

This exercise is directed toward interns and junior and senior residents.

**Time Required for Implementation:**

- Low-fidelity model preparation: Instructors should set aside 30 minutes per model for model assembly. This will need to take place prior to the session.
- Simulation preparation: Instructors should set aside 15 minutes before the start of each session in order to set the models in place, prepare the equipment, set up the PowerPoint presentation and quiz (Kahoot™).
- Training package delivery: 40–50 minutes will be required to run the simulation training package
- Debriefing: 5–10 minutes

**Recommended Number of Learners per Instructor:**

4–6

**Topics:**

Simulation, emergency medicine, orbital compartment syndrome, lateral canthotomy, cantholysis, procedural skills, low-fidelity model.

**Objectives:**

By the end of this simulation, learners should be able to:

1. Identify key clinical signs of OCS and explain the indications for LCC.
2. Prepare equipment and the procedural field for the LCC procedure.
3. Perform the step-by-step procedure safely on a low-fidelity model.

### Linked objectives, methods and results

#### Objective 1 - *Identify key clinical signs of OCS and explain the indications for LCC.*

The simulation package starts with an instructor-led presentation on OCS, outlining key indications, the clinical presentations, aetiology of the conditions as well as a video of the procedure being performed in clinical practice. The presentation outlines which key signs are required to make a clinical diagnosis in order to proceed with performing LCC, as well as contra-indications to performing the procedure. The instructor will discuss ways in how to approach a patient examination when assessing for OCS and which specific signs need to be assessed. A key point for learners to acknowledge is that OCS is a clinical diagnosis and one that should be made quickly. Knowledge was consolidated in the form of a gamified quiz (Kahoot™) which was a method of teaching we found significantly improved learner engagement. The concept of gamification of learning is widely supported in its use in education, with it being a highly useful tool in knowledge retention, engagement, and satisfaction.^6^–^8^

#### Objective 2 - *Prepare equipment and the procedural field for the LCC procedure.*

Once the presentation and quiz were completed, learners would then be able to practice the canthotomy and cantholysis procedures in a simulated environment with use of the low fidelity models. This would start by ensuring all learners are provided with the necessary equipment to perform the procedure, and that they can identify all the equipment required to perform the procedure safely within a simulated environment.

#### Objective 3 - *Perform the step-by-step procedure safely on a low-fidelity model.*

All learners were provided with physical handouts to help guide their procedures, in addition to circulating instructors offering their support. The authors created a video, shown after the presentation, highlighting how to perform the procedure on our low-fidelity models. Following the video and quiz to aid further consolidation, we found learners did not have to refer to their handouts often to complete the procedure step by step but found it a useful reference for ensuring their steps were correct and as a reference.

### Recommended pre-reading for instructor

- RCEM 2021 Curriculum (procedural skills section): The Royal College of Emergency Medicine. EM Curriculum 2021. Updated August 04, 2021. Accessed June *2025.* At: https://rcem.ac.uk/em-curriculum/
- Desai NM, Shah S. Lateral Orbital Canthotomy. In: StatPearls. Treasure Island (FL): StatPearls Publishing; 2025. Available from*:* https://www.ncbi.nlm.nih.gov/books/NBK557476/
- Instructor PowerPoint presentation (Appendix A)
- Procedural video via YouTube (free video): www.youtube.com/watch?v=VBg0ST7upkg&ab_channel=LarryB.Mellick%2CMD

### Learner responsible content (LRC)

- Pre-reading is optional, but we recommend learners are aware of the RCEM curriculum section on ophthalmologic emergencies and understand their curriculum requirements. Learners should also review the following in order to familiarize themselves with the procedure and its indications:
- Review article: Orbital Compartment Syndrome: McCallum E, Keren S, Lapira M, Norris JH. Orbital compartment syndrome: an update with review of the literature*. Clin Ophthalmol.* 2019 Nov 7;13:2189–2194. doi:10.2147/OPTH.S180058

### Implementation Methods

Instructors can adapt this simulation package to any suitable learner level, from medical students to senior emergency medicine resident doctors. Instructors should begin with a brief 20-minute presentation on OCS (Appendix A). We would recommend collecting pre-intervention feedback (Appendix D) before starting the presentation which can be performed by completing a physical feedback form or an online form, which we used, allowing learners to scan a QR code before start of the presentation. Ratings were carried out using a 10-point Likert scale for both knowledge (1 = very poor, 5 = neutral, and 10 = very good) and confidence (1 = not confident at all, 5 = neutral, and 10 = very confident). This enabled comparison of learners’ confidence and competence scores pre-and post-delivery of the simulation package (Appendix E). Ratings were also carried out for all educational components of the course using a 10-point Likert scale (1 = not useful, 10 = very useful, Appendix E).

Following delivery of the presentation, learners should be encouraged to clarify any questions they may have regarding the procedure so that instructors can help them. We recommend showing a YouTube video after this, to show the LCC procedure being performed in clinical practice. Instructors may then consider adding in a multiple choice-style quiz to further learner engagement and consolidate their knowledge. We recommend a gamified quiz that can aid healthy engagement and competition among peers, such as Kahoot,™ an online quiz platform (Appendix B).

Once the presentation and quiz are completed, learners will then be shown a video of how to perform the LCC on the lowfidelity simulation models, designed by the authors, and finally given the opportunity to practice the canthotomy and cantholysis procedures in a simulated environment with use of the low fidelity models. We recommend all learners are provided with physical handouts to help guide their procedures (Appendix C), in addition to circulating instructors offering their support.

To finish the training session, we recommend a 5–10 minute debrief on summarizing key take-home points, as well as giving learners an opportunity to ask any questions and share their thoughts on their experiences. It would also be useful at this point to collect any post-intervention feedback (Appendix E) via the use of physical or online feedback forms in order to assess whether any improvements were identified in learners’ confidence and competence scores following the session.

### List of items required to replicate this innovation

The items required for construction of the simulation model include the following:

- Standard size table tennis ball
- 10 ml box container
- Pressure foam tape
- Micropore surgical tape
- Transpore tape 3M
- Elastoplast fabric strapping tape
- Elastic rubber band
- Cardboard pieces 5.2 cm x 5.2 cm (x5)
- Duct tape
- Scissors
- Box Cutter
- Craft knife
- Marking pen

### Approximate cost of items to create this innovation

The equipment required for each model involves: 1) 6.5 cm × 6.5 cm food container - $0.50 ($5.50 for a set of 12), 2) 3M Microfoam tape $0.50 ($15.10 for 3 metres), 3) 3M Micropore/Transpore tape - $ 0.05 ($3.50 for 10 yards), 4) duct tape (single roll) - $0.03 ($9.45 for 45 yards), 5) cardboard (sheet/small box) - $1.00, 6) rubber bands (6 mm width) - $0.10, and 7) ping pong ball - $0.50. Total approximate cost for each model would be $2.68. A Stanley knife or craft knife may also be required ($8.00).

### Detailed methods to construct this innovation

**Figure 1:**
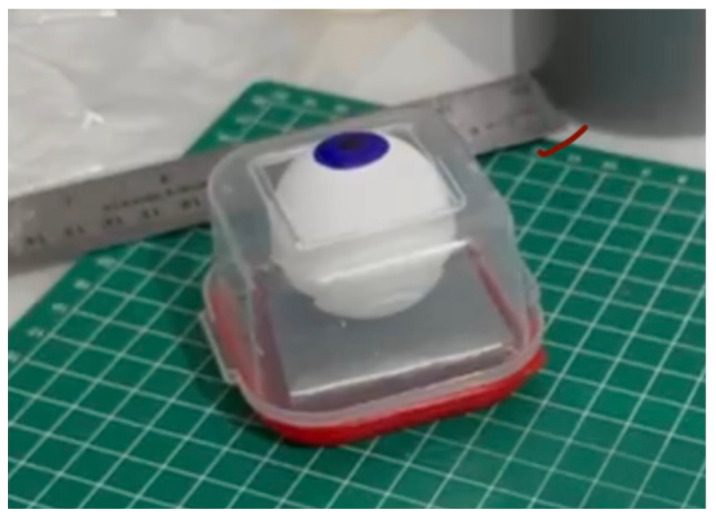
Step 1 of assembly

**Figure 2:**
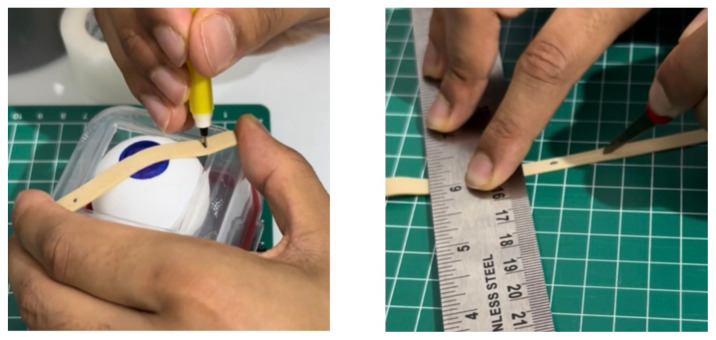
Step 2 of assembly

**Figure 3:**
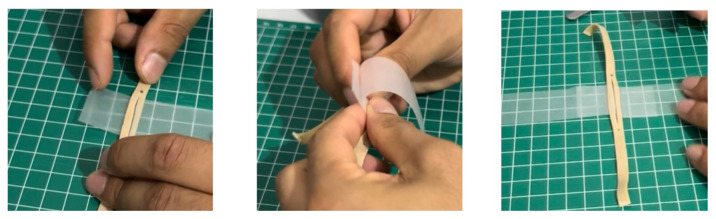
Step 3 of assembly

**Figure 4:**
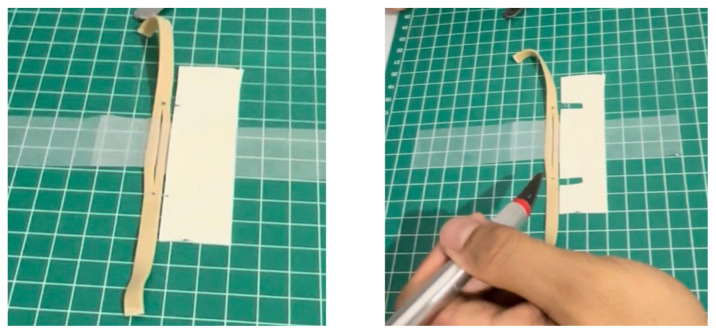
Step 4 of assembly

**Figure 5:**
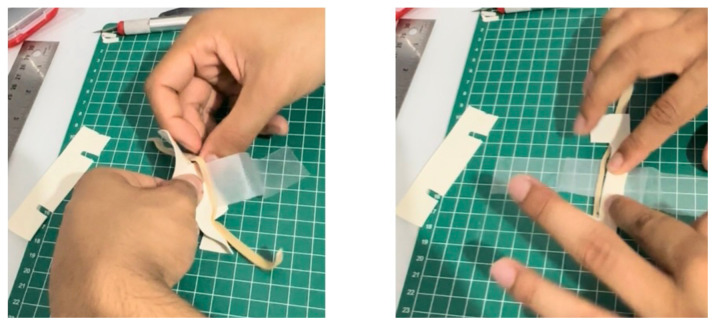
Step 5 of assembly

**Figure 6:**
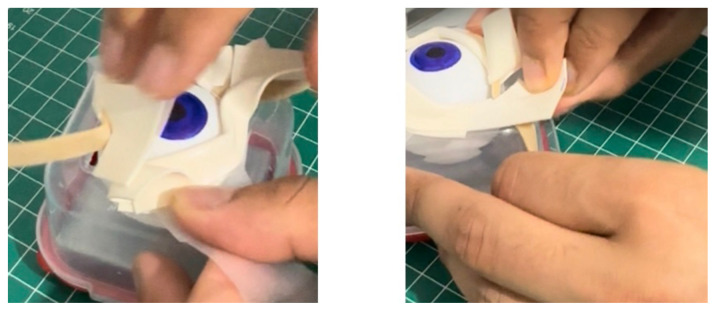
Step 6 of assembly

**Figure 7:**
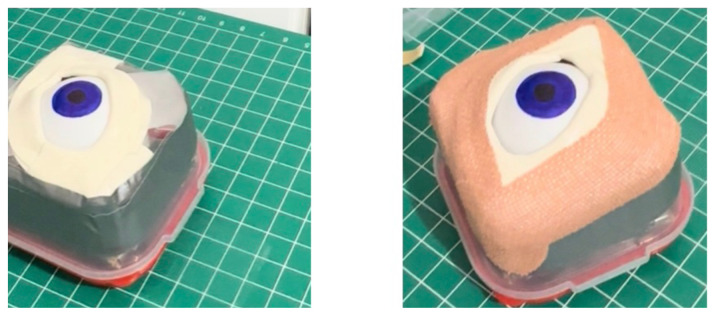
Step 7 of assembly

1. Using a box cutter, cut out a rectangular shaped hole from the base of the container, and insert a table tennis ball into the container so that it is in contact with the rectangular shaped hole. Cut five cardboard pieces (5.2 cm x 5.2 cm), layer them on top of each other in a stack, and secure the stack together with duct tape. Use this stack, placing it under the table tennis ball, to support it in place within the container (Fig. 1)
2. Cut a circular elastic band at any point to create a straight elastic band. Stretch the medial third of the elastic band gently over the face of the container with the rectangular hole, so that it covers two diagonal corners of the rectangular hole in a straight line. Using a pen, mark out the points at which the elastic band exactly overlies the diagonal corners (Fig. 2A). Following this, using a craft knife, bisect the elastic band lengthwise in the middle between these two points to simulate the lateral canthus superior and inferior tendons (Fig. 2B).
3. Cut two pieces of microspore tape of 8 cm length. Enter each piece approximately 2 cm through the medial bisection (Fig, 3A), folding the tape pieces at this point away from the bisection (Fig. 3B), both superiorly and inferiorly to the elastic band bisection (Fig. 3C).
4. Cut two pieces of foam tape of 9 cm length. Approximate this with the bisected segment of the elastic band so that the bisected segment lines up with the medial portion of the foam tape pieces. Using a pen, mark the lateral points of the bisected segment on the foam tape pieces (Fig. 4A). Using a craft knife, cut out two small flaps half the width of the foam tape pieces, approximately 0.5 cm apart (Fig. 4B). Repeat this process so that it is completed for both the superior and inferior portions of the bisected elastic band segment.
5. Place the foam tape pieces on the superior and inferior portions of the elastic band, so that both flaps on the foam tape overhang the lateral edges of the bisected elastic band segment (Fig. 5A). Then fold the foam tape pieces at the midline over the rubber band, completing this for both the superior and inferior portions of the elastic band (Fig. 5B).
6. Place the superior and inferior microspore tape pieces and tape them to the container so that the foam tape pieces lie superior and inferior to the pupil of the simulated eyeball (Fig. 6A). Tape the foam tape pieces to the container, with the lateral flaps of the foam tape folded down onto the container in alternate order (Fig. 6B).
7. Cut off any excess tape from the sides of the container. Use duct tape to surround the sides of the container and to support the lateral edges of the foam tape pieces (Fig. 7A). For the simulation of skin, apply elastoplast fabric strapping tape superior and inferior to the foam tape pieces, on the superior and inferior segments of the orbit, ensuring not to cover the areas of canthotomy incisions (Fig. 7B).

### Training delivery

For the simulation, the following equipment is required:

- Syringe with 1–2 ml of saline or local anaesthetic with adrenaline, attached with a 25-gauge needle
- Pair of toothed forceps
- Needle holder or haemostat
- Pair of iris scissors or suture scissors

The following steps are required for delivery of the training session:

1. Positioning the needle away from the globe, aspirate initially and inject 1–2 ml of saline or local anaesthetic with adrenaline into the lateral canthus. Dispose of the needle following administration into a sharps bin.
2. Using the haemostat/needle holder, clamp the lateral canthus tissue ensuring that the inferior jaw of the needle holder/haemostat contacts the bony orbital ring. Once contact is established, the tissue can be clamped for a period of 20 – 60 seconds.
3. Using a pair of iris or suture scissors, cut along the clamped area of tissue, making a 1–2 cm incision.
4. Using a pair of toothed forceps, retract the lateral portion of the inferior eyelid to visualize the lateral canthal tendon. Using iris or suture scissors, strum around the area to identify the position and integrity of the inferior crus of the lateral canthal tendon, using tactile feedback.
5. Using a pair of iris or suture scissors, cut the inferior crus of the lateral canthal tendon in the inferoposterior direction, cutting away from the globe to avoid injury to the globe or surrounding structures.
6. For confirmation of cantholysis, use a pair of toothed forceps to apply inferior traction to the lateral portion of the inferior eyelid, to ensure the inferior crus is cut.
7. Repeat the above steps for the superior crus of the lateral canthal tendon, on the lateral portion of the superior eyelid. Ensure that during cantholysis, the incision is made in the superoposterior direction to avoid injury to the globe and surrounding structures.

### Results and tips for successful implementation

This simulation was performed in the simulation suite at King’s College Hospital, London, from 2022 to 2023, Basildon & Thurrock University Hospital in August 2023, and Southend University Hospital in October 2023, as part of mandatory comprehensive simulation skills days delivered annually for regional emergency medicine trainees within their respective deaneries.

Data were analyzed at both cohort and combined levels. Descriptive statistics (mean ± standard deviation) were calculated for all groups. To compare pre- and post-session scores within each cohort and overall, paired t-tests were performed to determine statistical significance, with a p-value of <0.05 considered significant. Qualitative feedback was analyzed to identify positive responses in addition to suggestions for improvement.

**Figure 8:**
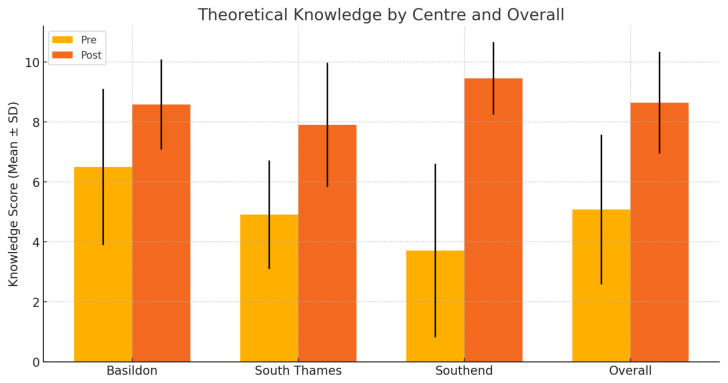
Bar graph comparing theoretical knowledge scores pre- and post-intervention

**Figure 9:**
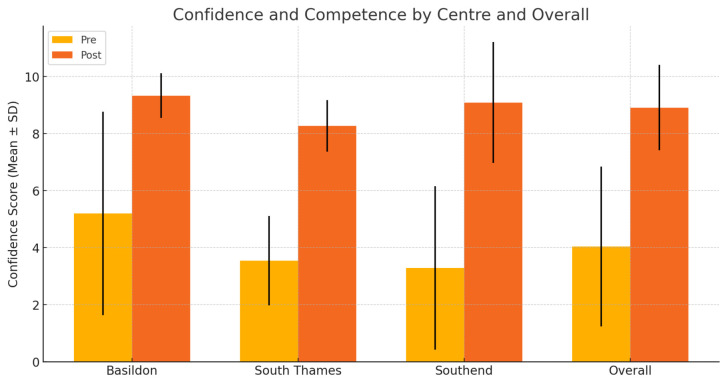
Bar graph comparing confidence and competence scores pre-and post-intervention

Across three UK training centres (n=34), participants reported marked gains in both confidence and knowledge. Mean knowledge scores demonstrated a significant increase following delivery of this teaching package (5.0 ± 2.5 to 8.7 ± 1.7; p < 0.0001) (Table 1, Figure 8). Similarly, confidence improved markedly, indicating further consistency across all cohorts (4.1 ± 2.8 to 8.9 ± 1.5; p < 0.0001) (Table 2, Figure 9).

All trainees consistently rated each element of the simulation training package highly, with mean scores ranging between 8.7 and 10 (Table 3). The PowerPoint presentations were particularly appreciated across all sites, rated between 9.0 ± 0.8 and 9.7 ± 0.6. The procedural video received scores from 9.2 ± 0.9 to 9.6 ± 0.9. The low-fidelity simulator was also highly valued, achieving mean scores of 9.4 ± 1.0 to 9.9 ± 0.3, and the multiple choice quiz ranged from 8.7 ± 1.1 to 10.0 ± 0, supporting its role in knowledge consolidation. Importantly, 100% of trainees across all centres indicated that they supported the annual delivery of this training simulation package for emergency medicine trainees.

On analysis of the qualitative feedback collected, the comments were overwhelmingly positive (Table 4). Of note, trainees stated an approval of the structure and clarity of the session, with particular emphasis on the usefulness of inclusion of the low-fidelity simulator in consolidating the knowledge learned.

### Associated Content

Appendix A: PowerPoint Presentation

Appendix B: Quiz Questions and Answers

Appendix C: Lateral Canthotomy and Cantholysis Procedural Handout (Step-by-Step Guide)

Appendix D: Pre-Intervention Feedback

Appendix E: Post-Intervention Feedback

## Supplementary Information



## Figures and Tables

**Table 1:** Theoretical knowledge scores pre- and post-intervention

Centre	n	Pre-intervention (mean ± Standard Deviation)	Post-intervention (mean ± Standard Deviation)	p-value
**Basildon**	12	6.5 ± 2.6	8.6 ± 1.5	0.038
**South Thames**	11	4.9 ± 1.8	7.9 ± 2.1	0.0008
**Southend**	11	3.5 ± 2.9	9.5 ± 1.2	0.0002
**Overall**	34	5.0 ± 2.5	8.7 ± 1.7	<0.0001

**Table 2:** Confidence and competence scores pre- and post-intervention

Centre	n	Pre-intervention (mean ± SD)	Post-intervention (mean ± SD)	p-value
Basildon	12	5.2 ± 3.6	9.3 ± 0.8	*See note below
South Thames	11	3.5 ± 1.6	8.3 ± 0.9	<0.0001
Southend	11	3.5 ± 3.0	9.1 ± 2.1	0.0016
Overall	34	4.1 ± 2.8	8.9 ± 1.5	<0.0001

* **Footnote:** *Due to a ceiling effect with very low variance in Basildon confidence and competence post-intervention score, a reliable p-value could not be calculated via paired t-tests. However, improvement was descriptively large.*

**Table 3:** Post-Intervention Ratings of Educational Components

Centre	n	Power Point (mean ± SD)	Video (mean ± SD)	Simulator (mean ± SD)	MCQ quiz (mean ± SD)	Annual delivery support
Basildon	12	9.0 ± 1.1	9.5 ± 0.6	9.9 ± 0.3	9.6 ± 0.7	100%
South Thames	11	9.0 ± 0.8	9.2 ± 0.9	9.5 ± 0.7	8.7 ± 1.1	100%
Southend	11	9.7 ± 0.6	9.6 ± 0.9	9.4 ± 1.0	10.0 ± 0	100%
Overall	34	9.2 ± 1.0	9.4 ± 0.8	9.6 ± 0.7	9.4 ± 0.8	100%

**Table 4:** Free Text Qualitative Feedback

Centre	Free Text Comment
Basildon	Very useful for training
Basildon	No comments, very good lesson
Basildon	Good models and presentation
South Thames	Anchoring the model in a pretend face
South Thames	Excellent session thanks!
South Thames	Great sim! The model has a great potential to be marketed as a teaching tool
South Thames	could add a sim element
South Thames	Really useful session thanks
Southend	Excellent
Southend	Excellent presenter. Keep the same presenter.
Southend	Absolutely amazing teaching. Very structured. Amazing effort. Well presented. Would definitely recommend it to colleagues.
Southend	In future, if possible, to use animal cadaver models.
Southend	No, it is just perfect
Southend	Excellent presentation

**Figure:**
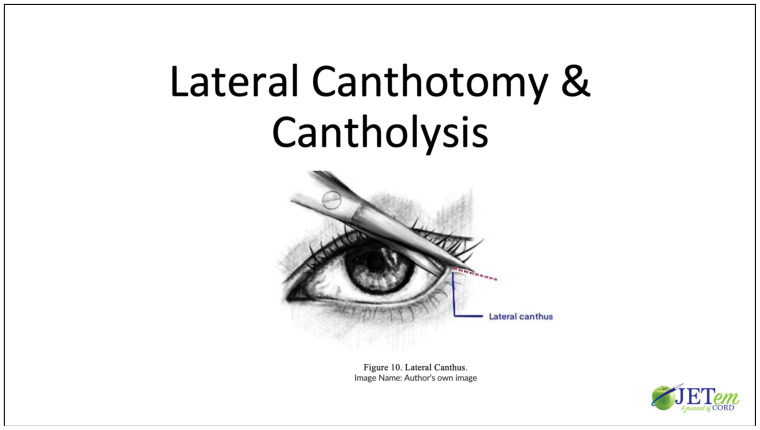
Please see associated PowerPoint file

## INNOVATION MATERIALS

### Appendix B Quiz Questions

1. The diagnosis for Orbital Compartment Syndrome is a...
  - Radiological diagnosis
  - Clinical diagnosis
  - Histological diagnosis
  - Serological diagnosis
2. Recent studies have shown that irreversible vision loss can occur after how long following the onset of retinal ischaemia?
  - 15 minutes
  - 30 minutes
  - 1 hour
  - 2 hours
  - 3 hours
3. For how long do you crush the lateral canthus tissue with the haemostat to achieve haemostasis?
  - 10 to 20 seconds
  - 20 seconds to 1 minute
  - 1 to 2 minutes
  - 5 minutes or more
4. In actual clinical practice it can be challenging to visualize the inferior crus; how can we confirm the position and integrity of the inferior crus?
  - Irrigate the surgical field with saline for a clearer view
  - Using a brighter light for a clearer view
  - Strumming the tendon to assess for tendon tension
  - Applying inferior traction to the lower eyelid using toothed forceps
5. To avoid injury to surrounding structures, in which direction should you cut the inferior crus of the lateral canthal tendon?
  - Superomedially and towards the globe
  - Inferiorposteriorly and away from the globe
  - Laterally and parallel to the globe
  - Inferiorly
6. Which of the following examination findings would contraindicate intervention with a lateral canthotomy and cantholysis?
  - Proptosis
  - Loss of visual acuity
  - Ophthalmoplegia
  - Hyphaema
  - Relative Afferent Pupillary Defect

#### Quiz Answers

1. The diagnosis for Orbital Compartment Syndrome is a...
  a. Radiological diagnosis
  **b. Clinical diagnosis**
  c. Histological diagnosis
  d. Serological diagnosis
    - NOTE: OCS is diagnosed clinically (based on Hx and examination). Never delay interventions to conduct other investigations
2. Recent studies have shown that irreversible vision loss can occur after how long following the onset of retinal ischaemia?
  **a. 15 minutes**
  b. 30 minutes
  c. 1 hour
  d. 2 hours
  e. 3 hours
    - NOTE: The time from the onset of OCS to the orbital decompression is the greatest prognostic indicator highlighting the need for rapid decisive action. This will enable restoration of perfusion to the optic nerve and retina.
3. For how long do you crush the lateral canthus tissue with the haemostat to achieve haemostasis?
  a. 10 to 20 seconds
  **b. 20 seconds to 1 minute**
  c. 1 to 2 minutes
  d. 5 minutes or more
4. In actual clinical practice it can be challenging to visualize the inferior crus; how can we confirm the position and integrity of the inferior crus?
  a. Irrigate the surgical field with saline for a clearer view
  b. Using a brighter light for a clearer view
  **c. Strumming the tendon to assess for tendon tension**
  d. Applying inferior traction to the lower eyelid using toothed forceps
    - NOTE: While the other measures listed can help visualize the tendon more clearly, they cannot provide you will the tactile feedback needed to determine whether the tendon has been lysed or not.
5. To avoid injury to surrounding structures, in which direction should you cut the inferior crus of the lateral canthal tendon?
  a. Superomedially and towards the globe
  **b. Inferiorposteriorly and away from the globe**
  c. Laterally and parallel to the globe
  d. Inferiorly
6. Which of the following examination findings would contraindicate intervention with a lateral canthotomy and cantholysis?
  a. Proptosis
  b. Loss of visual acuity
  c. Ophthalmoplegia
  **d. Hyphaema**
  e. Relative Afferent Pupillary Defect
    - NOTE: This is a sign of potential globe rupture which is a contraindication for the lateral canthotomy and cantholysis procedure. It would be essential to seek prompt specialist consultation in these cases.

## LEARNER MATERIALS

### Appendix C Lateral Canthotomy and Cantholysis Procedural Handout (Step-by-Step Guide)

#### Equipment

- Sterile gloves
- Antiseptic solution eg, chloraprep
- Surgical drape
- Local anaesthetic (1–2 ml with adrenaline)
- Haemostat or Needle holder*
- Toothed forceps*
- Iris scissors or Suture scissors*
- Sterile gauze*

*All found in the Fine Suture Pack

##### Step-by-Step Guide

1. Quick examination of visual acuity, the globe, and the lateral canthus area.
2. Irrigate the lateral canthus and surrounding tissue if necessary.
3. Cleanse the lateral canthus area with an antiseptic agent; be careful not to let any get into the eye.
4. Apply a surgical drape around the affected eye.
5. Inject 1–2 mL of local anaesthetic with adrenaline into the planned incision site; aim the tip of the needle AWAY from the globe.
  ○ Must ASPIRATE before injecting the local anaesthetic.
6. Use the haemostat to crush the tissue from the lateral canthus to the rim of the orbit (you should feel the bony orbital rim) and lock the haemostat in place for 20 secs to 1 min.
7. Remove the haemostat.
8. Using the iris scissors to conduct the canthotomy by cutting along the crushed tissue from the lateral canthus to the rim of the orbit. The incision should be no more than 2 cm in length.
9. Using the toothed forceps, apply inferior traction to the lateral portion of the lower eyelid to visualize the lateral canthal tendons.
10. Using the iris scissors, “strum” around the area to identify the position and tension of the inferior crus.
11. If the inferior crus tension is intact, using the iris scissors conduct the cantholysis by cutting the inferior crus; do this step INTERIOPOSTERIORLY to the globe to avoid injuring surrounding structures.
12. Reassess the patient - measure IOP.
  ○ NB: Vision should return/improve within 15 minutes to 6 hours post-procedure.
13. If IOP remains elevated, consider cutting the superior crus of the lateral canthus tendon.

##### Referral and Aftercare

- This is dependent on your hospital/trust
- Contact the on-call ophthalmology and/or MaxFax team (available 24/7)
  ○ Definitive treatment for the underlying cause of OCS is managed by ophthalmology
  ○ Facial trauma/fracture is managed by MaxFax
  ○ In trauma cases, you would refer to both teams
- In all cases of OCS, EARLY referral is essential because the lateral canthotomy and cantholysis may not successfully reduce the IOP in which case further interventions will be needed

### Appendix D Pre-Intervention Feedback

* Indicates required question

1. What has been your current level of exposure to lateral canthotomy and cantholysis procedures?*
  ○ Performed the procedure in real clinical practice
  ○ Simulated practice with an artificial model
  ○ Simulated practice with a cadaveric model
  ○ e-Learning
  ○ None
  ○ Other:
2. If you chose ‘Other’ for the previous question, please provide some brief details
3. Rate your theoretical knowledge of the lateral canthotomy and cantholysis procedure?*
  **Table:**
  1	2	3	4	5	6	7	8	9	10
  Very poor									Very good
4. Rate your confidence and competence in performing lateral canthotomy and cantholysis in real clinical practice?*
  **Table:**
  1	2	3	4	5	6	7	8	9	10
  Not confident at all									Very confident
5. What do you hope to gain most from this session?*

### Appendix E Post-Intervention Feedback

*Indicates required question

1. Rate your theoretical knowledge of the lateral canthotomy and cantholysis procedure AFTER the course?*
  **Table:**
  1	2	3	4	5	6	7	8	9	10
  Very poor									Very good
2. Rate your confidence and competence in performing lateral canthotomy and cantholysis in real clinical practice AFTER the course?*
  **Table:**
  1	2	3	4	5	6	7	8	9	10
  Not confident at all									Very confident
3. How useful did you find the PowerPoint presentation in improving or refreshing your theoretical knowledge?*
  **Table:**
  1	2	3	4	5	6	7	8	9	10
  Not useful at all									Very useful
4. How useful did you find the video in improving your procedural knowledge and skill?*
  **Table:**
  1	2	3	4	5	6	7	8	9	10
  Not useful at all									Very useful
5. How useful did you find the low-fidelity simulator in improving your procedural knowledge and skill?*
  **Table:**
  1	2	3	4	5	6	7	8	9	10
  Not useful at all									Very useful
6. How useful did you find the multiple-choice quiz in consolidating your theoretical and procedural knowledge?*
  **Table:**
  1	2	3	4	5	6	7	8	9	10
  Not useful at all									Very useful
7. Do you think this training package should be delivered annually as part of the EM trainee training?*
  ○ Yes
  ○ No
8. Do you have any suggestions regarding improvements that can be made to this training package?

## References

[1] McCallum E, Keren S, Lapira M, Norris JH (2019). Orbital compartment syndrome: an update with review of the literature. Clin Ophthalmol.

[2] Sotomayor TM, Bailey MP, Dorton SL (2019). Using simulation to address a training gap in battlefield ocular trauma: a lateral canthotomy and cantholysis (LCC) prototype training system. Mil Med.

[3] Weightman J, Latham K, Bowyer MW, Andreatta P (2024). Lateral canthotomy/cantholysis performance gap analysis and training recommendations for expeditionary physicians. Mil Med.

[4] Kong MC, Knights R, Johnston MJ, Gibbs D (2018). Development of a novel low fidelity model for training in lateral canthotomy. Clin Teach.

[5] Issenberg SB, McGaghie WC, Petrusa ER, Gordon DL, Scalese RJ (2005). Features and uses of high-fidelity medical simulations that lead to effective learning: a BEME systematic review. Med Teach.

[6] van Gaalen AE, Brouwer J, Schönrock-Adema J, Bouwkamp-Timmer T, Jaarsma ADC, Georgiadis JR (2021). Gamification of health professions education: a systematic review. Adv Health Sci Educ Theory Pract.

[7] Borges NJ, Manuel RS, Elam CL, Jones BJ (2010). Differences in motives between Millennial and Generation X medical students. Med Educ.

[8] Gentry SV, Gauthier A, Ehrström BL (2019). Serious gaming and gamification education in health professions: systematic review. J Med Internet Res.

