# Supplementary material for: A Multimodal Approach to Lateral Canthotomy and Cantholysis Training for Emergency Medicine Trainees: A Simulation Training Package
Source: J Educ Teach Emerg Med. 2026 Jan 31;11(1):I1–I18. doi: 10.5070/M5.52351 (PMC12880893; doi:10.5070/M5.52351)
Supplement: Supplementary file 1 [file Appendix_A.pptx]

## Slide 1
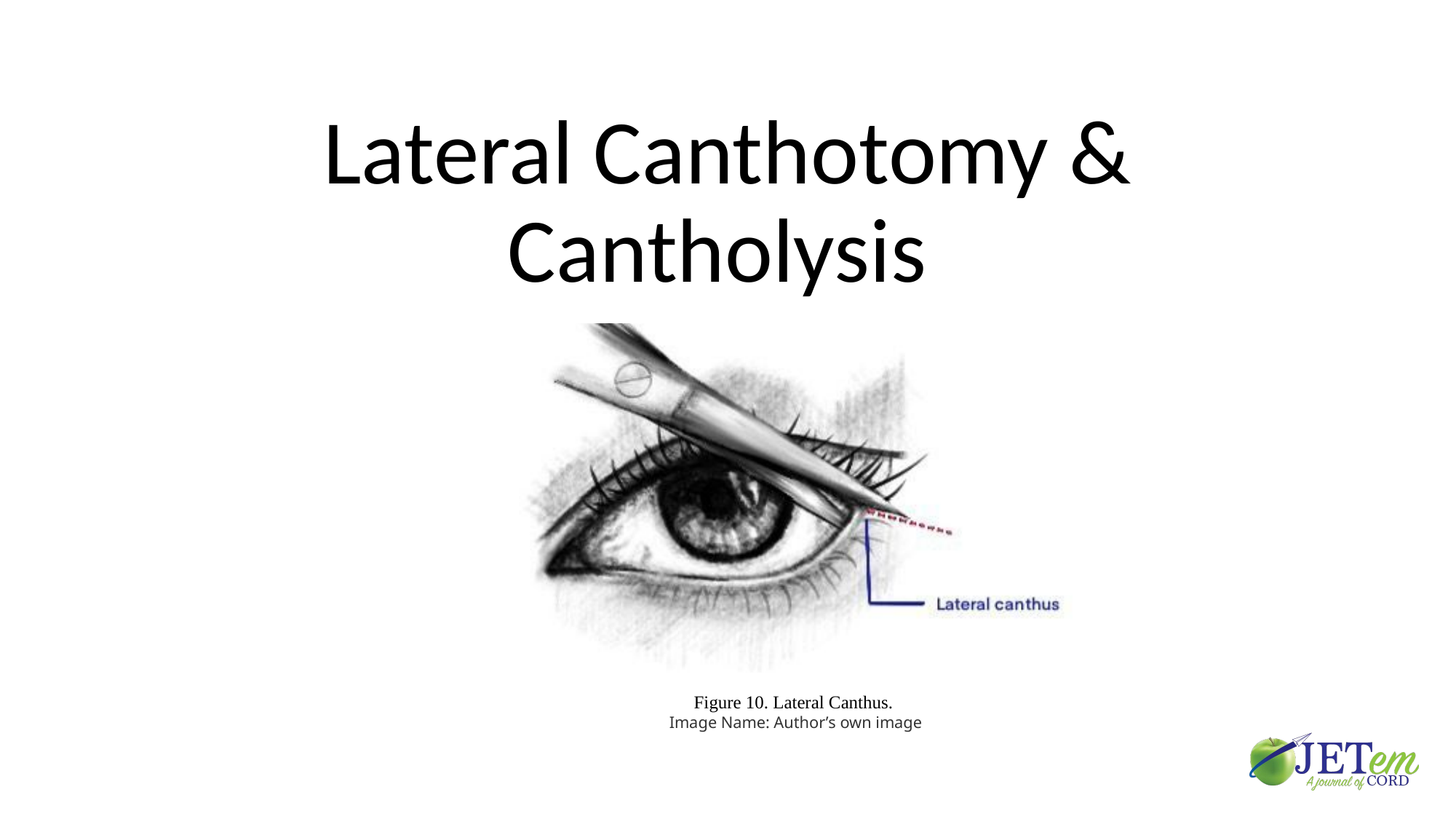

# Lateral Canthotomy & Cantholysis
Figure 10. Lateral Canthus.
Image Name: Author’s own image

## Slide 2
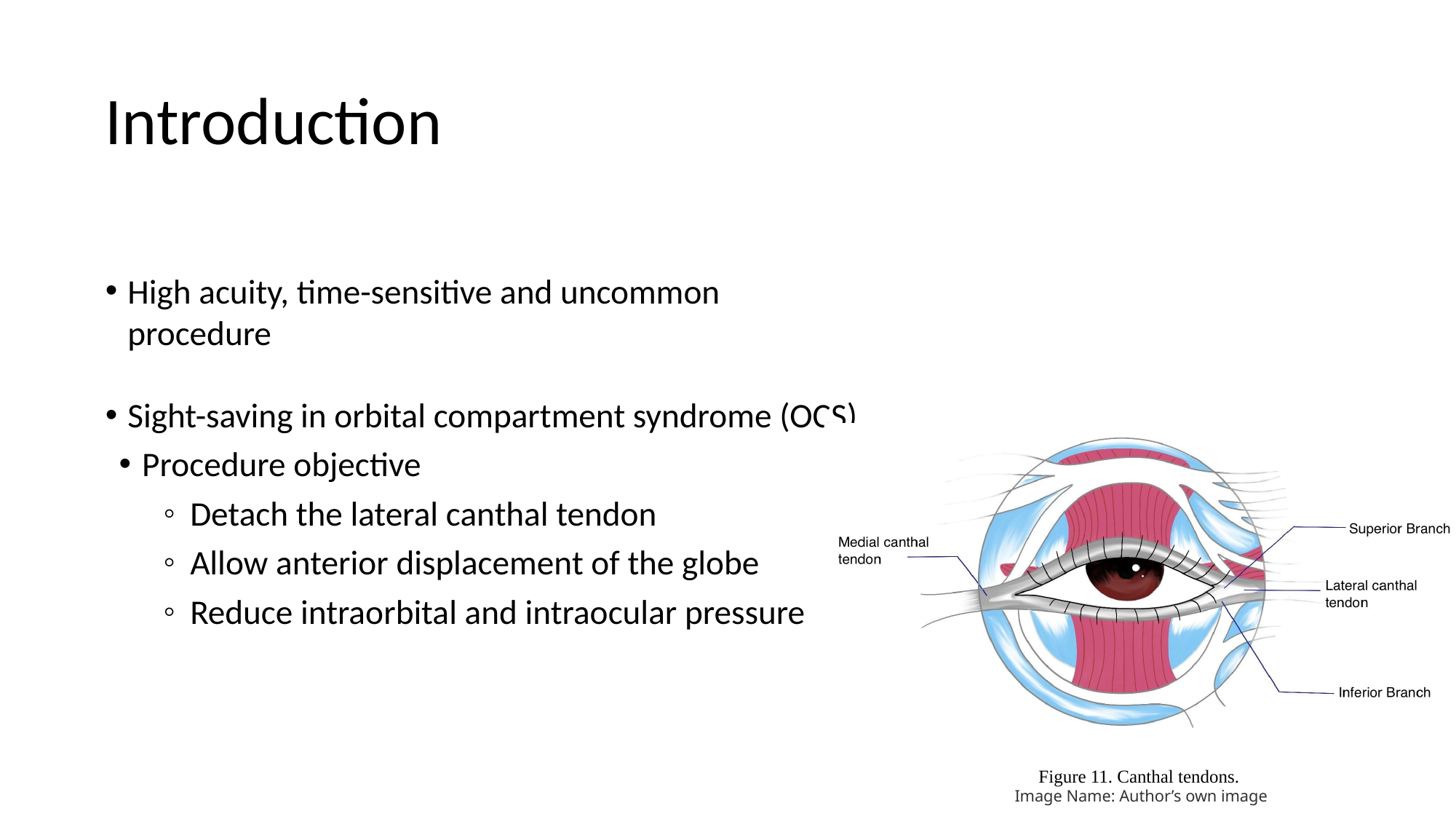

# Introduction
High acuity, time-sensitive and uncommon procedure
Sight-saving in orbital compartment syndrome (OCS)
Procedure objective
Detach the lateral canthal tendon
Allow anterior displacement of the globe
Reduce intraorbital and intraocular pressure
Figure 11. Canthal tendons.
Image Name: Author’s own image

## Slide 3
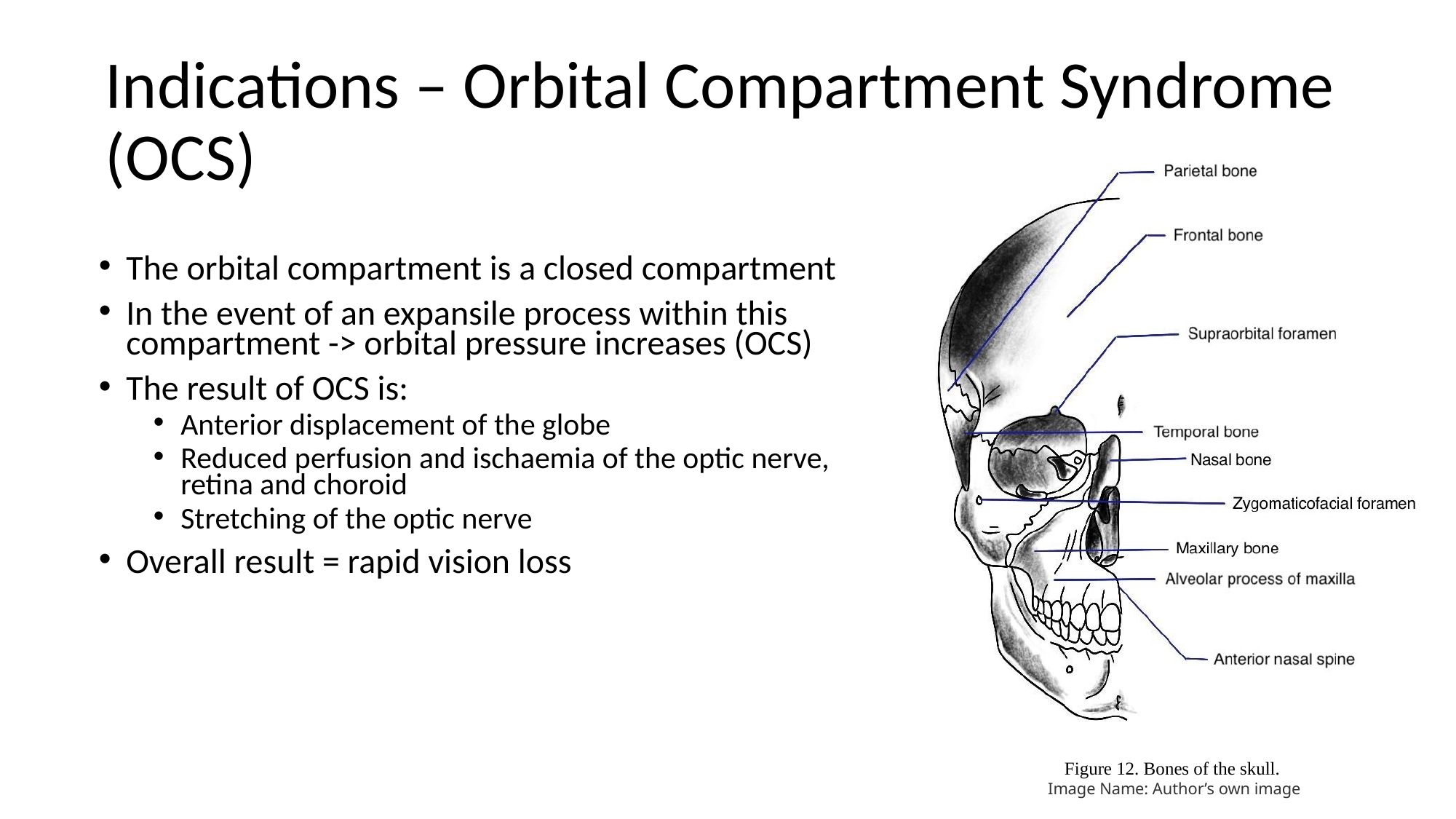

# Indications – Orbital Compartment Syndrome (OCS)
Figure 12. Bones of the skull.
Image Name: Author’s own image
The orbital compartment is a closed compartment
In the event of an expansile process within this compartment -> orbital pressure increases (OCS)
The result of OCS is:
Anterior displacement of the globe
Reduced perfusion and ischaemia of the optic nerve, retina and choroid
Stretching of the optic nerve
Overall result = rapid vision loss

## Slide 4
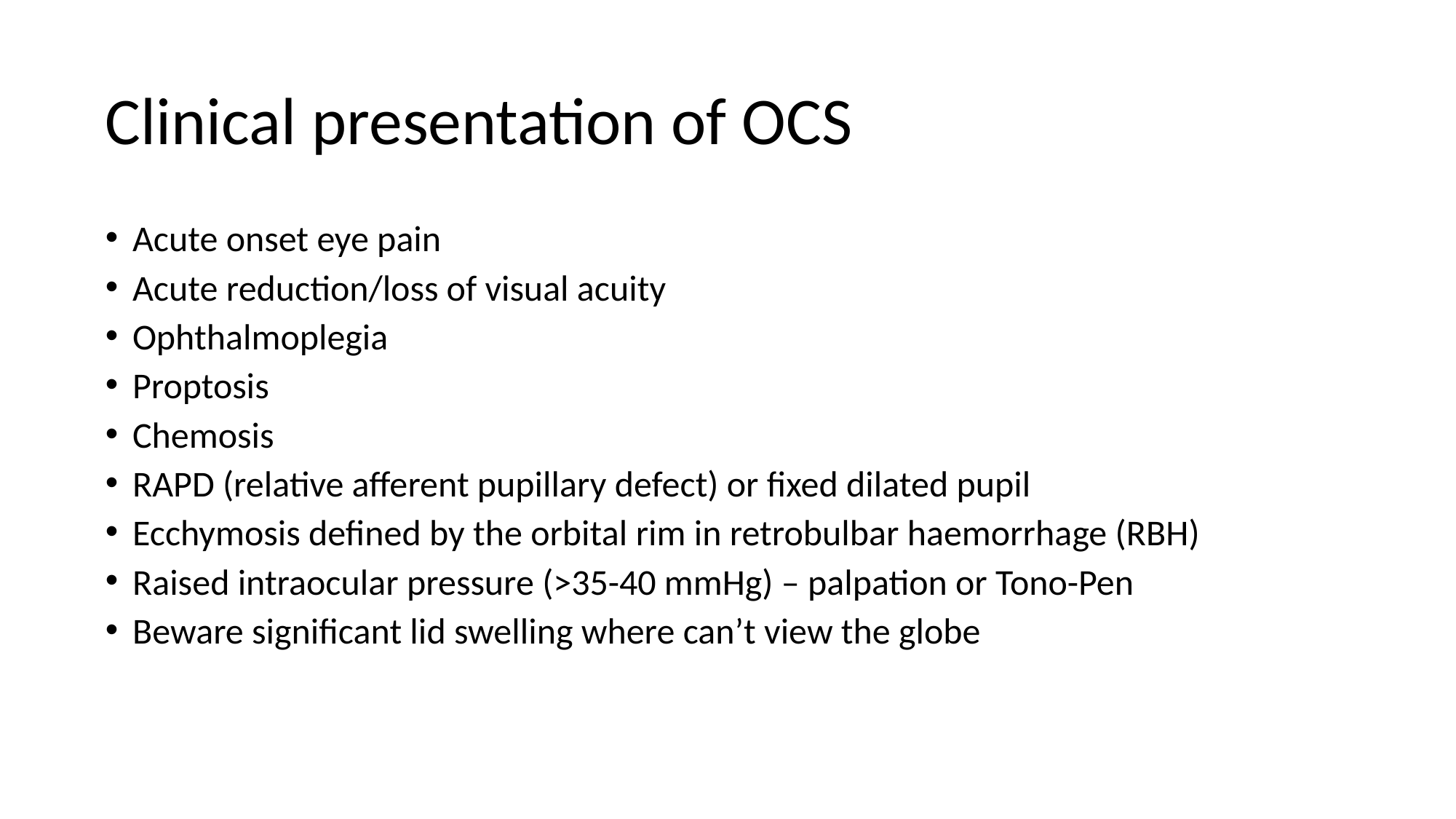

# Clinical presentation of OCS
Acute onset eye pain
Acute reduction/loss of visual acuity
Ophthalmoplegia
Proptosis
Chemosis
RAPD (relative afferent pupillary defect) or fixed dilated pupil
Ecchymosis defined by the orbital rim in retrobulbar haemorrhage (RBH)
Raised intraocular pressure (>35-40 mmHg) – palpation or Tono-Pen
Beware significant lid swelling where can’t view the globe

## Slide 5
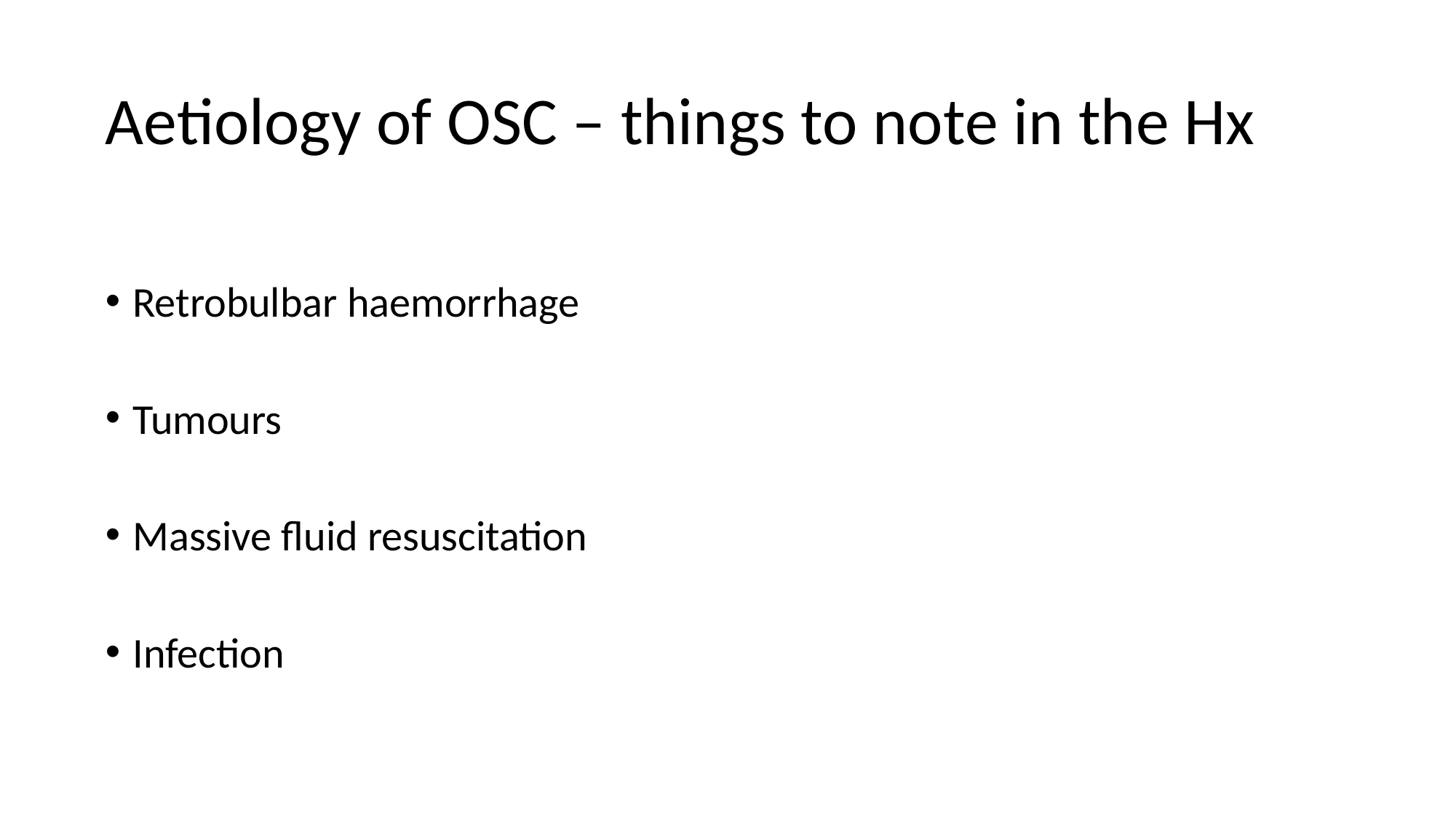

# Aetiology of OSC – things to note in the Hx
Retrobulbar haemorrhage
Tumours
Massive fluid resuscitation
Infection

## Slide 6
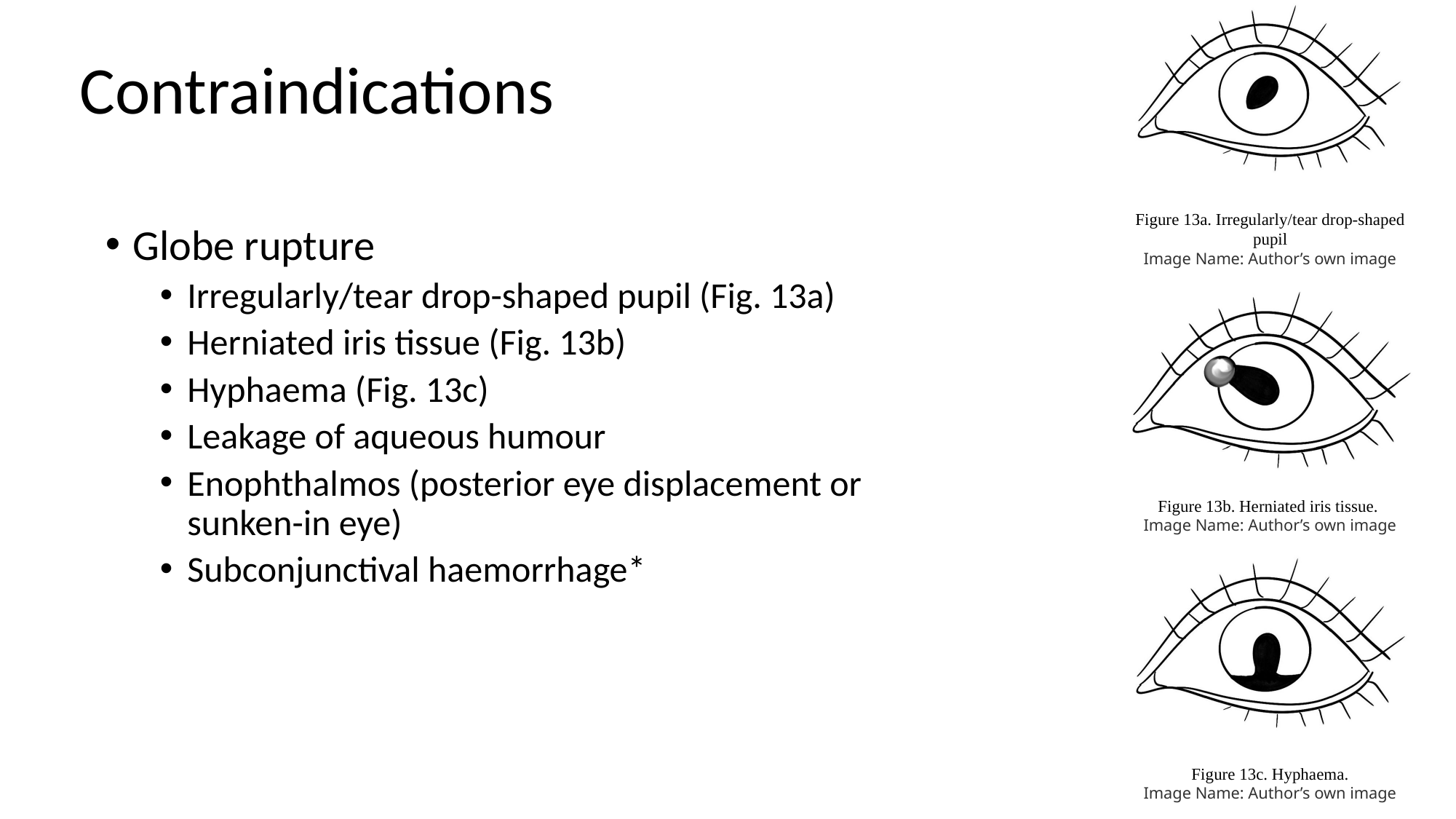

Figure 13a. Irregularly/tear drop-shaped pupil
Image Name: Author’s own image
# Contraindications
Globe rupture
Irregularly/tear drop-shaped pupil (Fig. 13a)
Herniated iris tissue (Fig. 13b)
Hyphaema (Fig. 13c)
Leakage of aqueous humour
Enophthalmos (posterior eye displacement or sunken-in eye)
Subconjunctival haemorrhage*
Figure 13b. Herniated iris tissue.
Image Name: Author’s own image
Figure 13c. Hyphaema.
Image Name: Author’s own image

## Slide 7
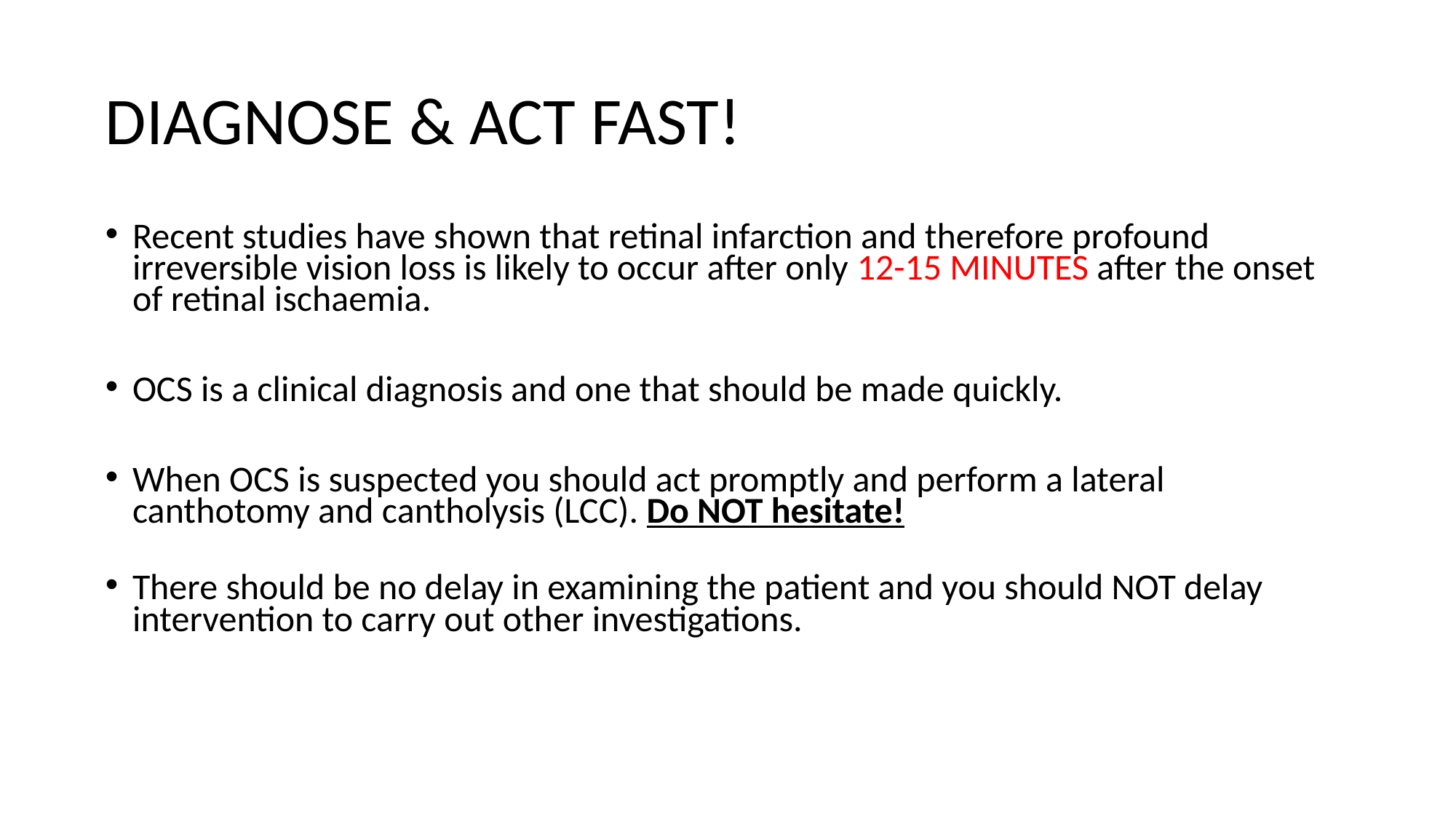

# DIAGNOSE & ACT FAST!
Recent studies have shown that retinal infarction and therefore profound irreversible vision loss is likely to occur after only 12-15 MINUTES after the onset of retinal ischaemia.
OCS is a clinical diagnosis and one that should be made quickly.
When OCS is suspected you should act promptly and perform a lateral canthotomy and cantholysis (LCC). Do NOT hesitate!
There should be no delay in examining the patient and you should NOT delay intervention to carry out other investigations.

## Slide 8
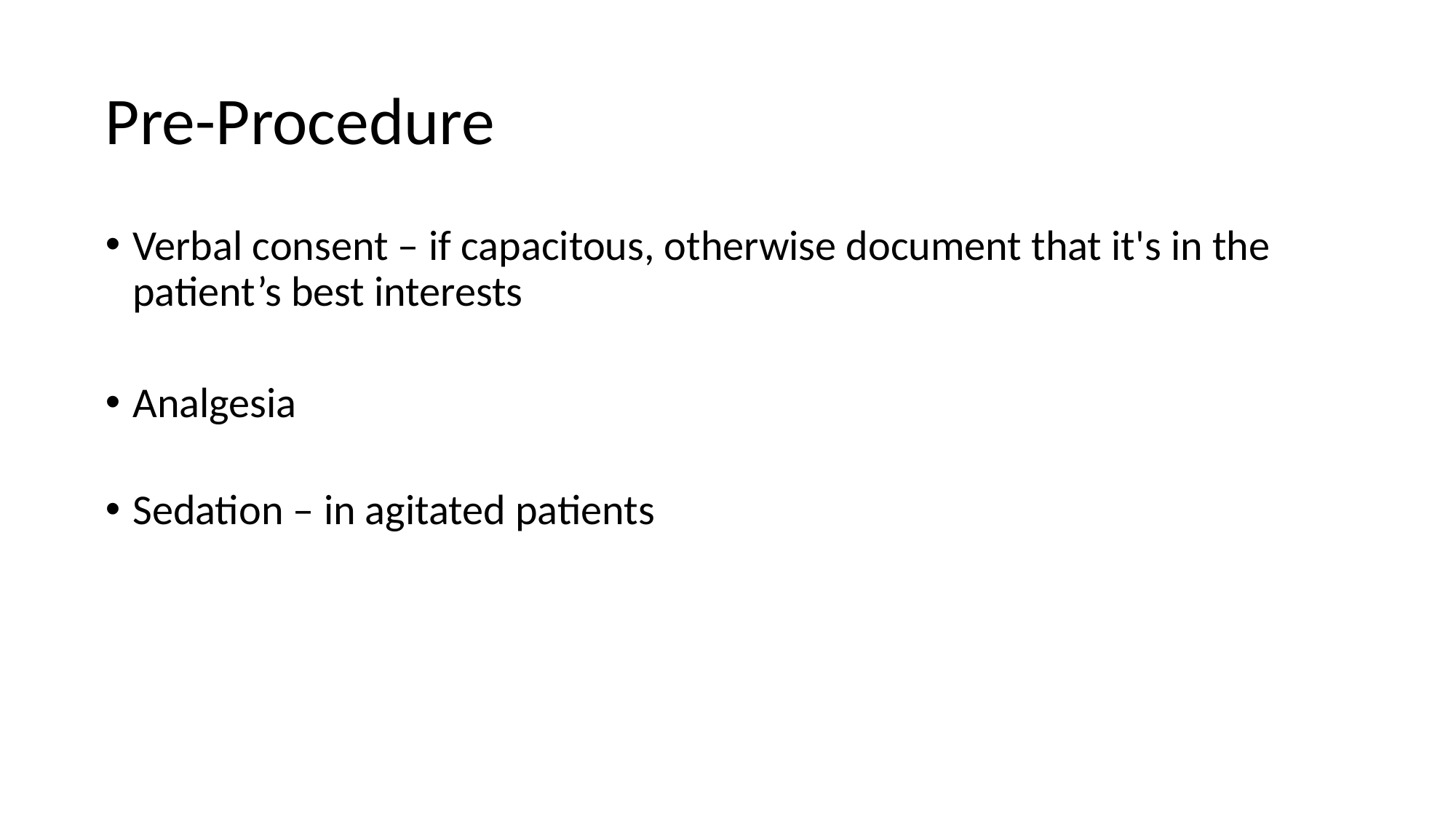

# Pre-Procedure
Verbal consent – if capacitous, otherwise document that it's in the patient’s best interests
Analgesia
Sedation – in agitated patients

## Slide 9
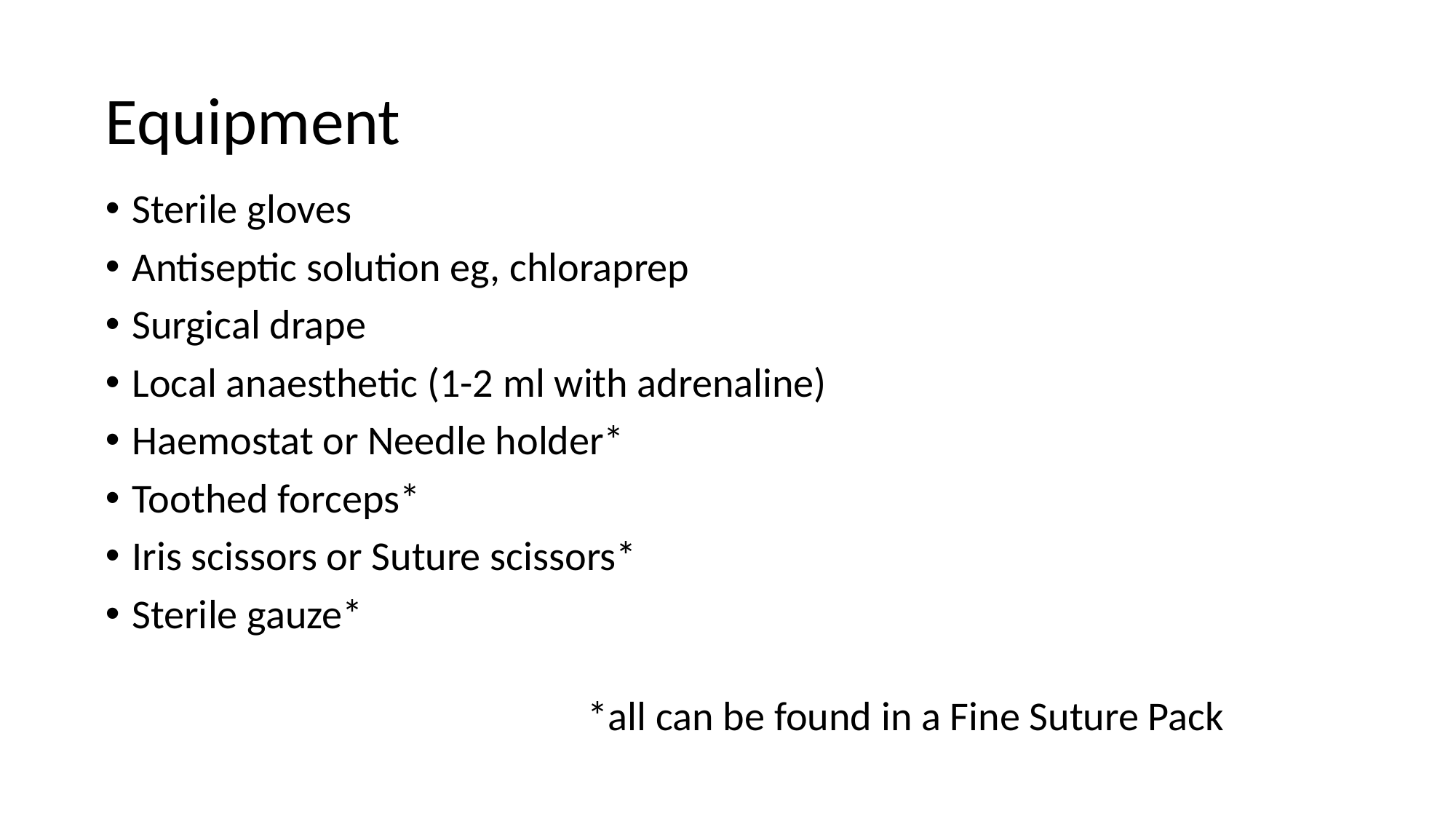

# Equipment
Sterile gloves
Antiseptic solution eg, chloraprep
Surgical drape
Local anaesthetic (1-2 ml with adrenaline)
Haemostat or Needle holder*
Toothed forceps*
Iris scissors or Suture scissors*
Sterile gauze*
 *all can be found in a Fine Suture Pack

## Slide 10
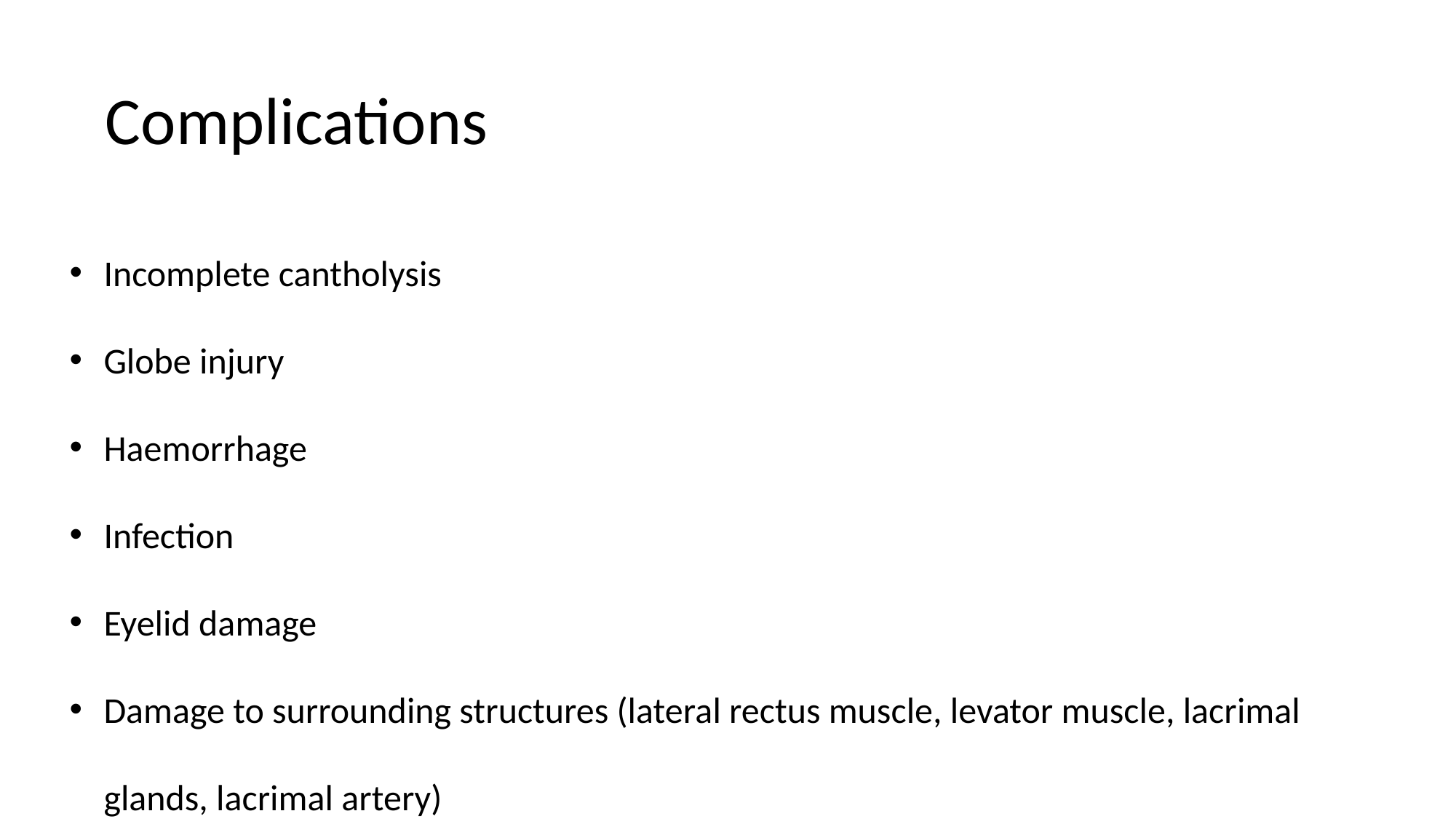

# Complications
Incomplete cantholysis
Globe injury
Haemorrhage
Infection
Eyelid damage
Damage to surrounding structures (lateral rectus muscle, levator muscle, lacrimal glands, lacrimal artery)

## Slide 11
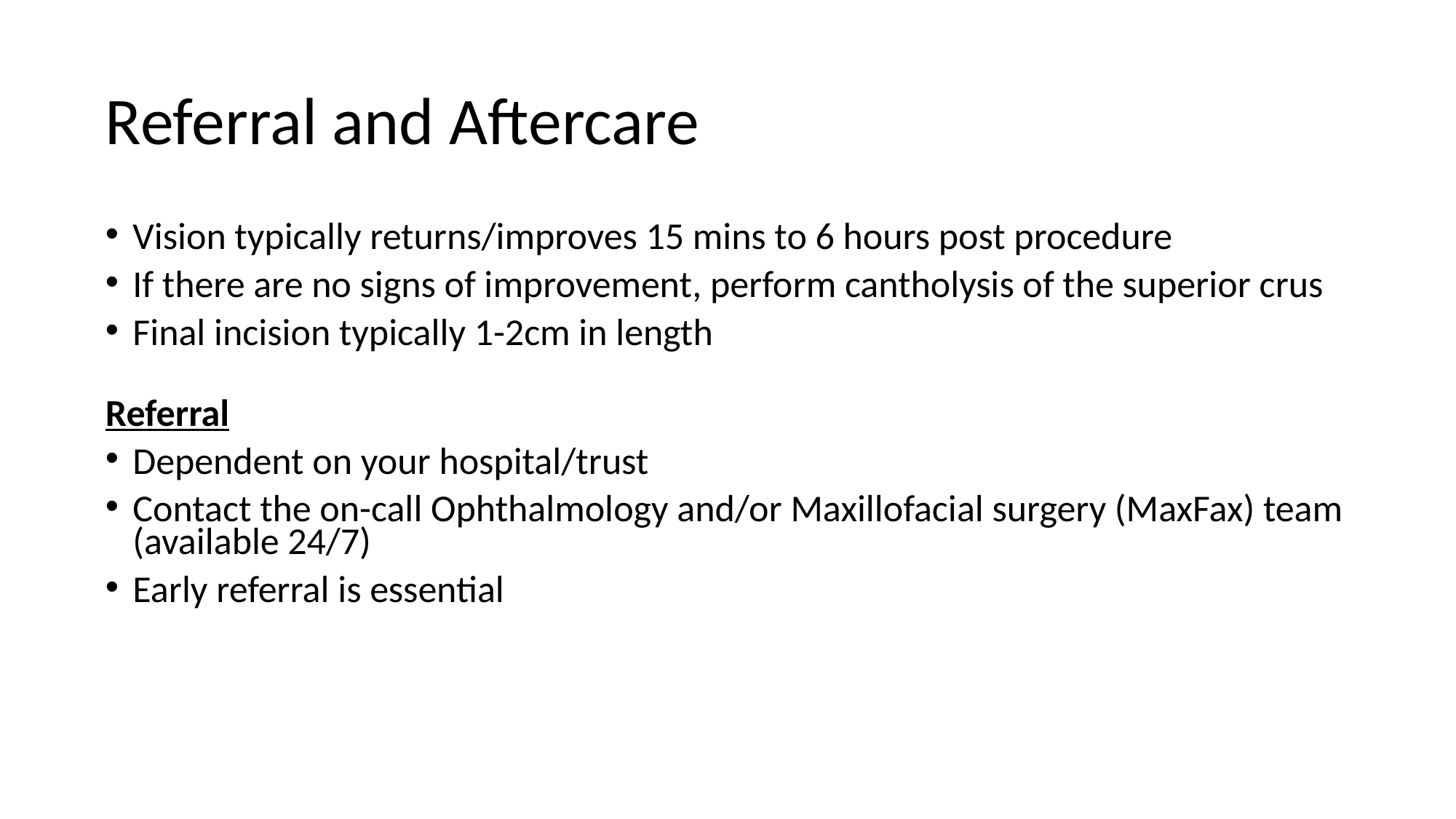

# Referral and Aftercare
Vision typically returns/improves 15 mins to 6 hours post procedure
If there are no signs of improvement, perform cantholysis of the superior crus
Final incision typically 1-2cm in length
Referral
Dependent on your hospital/trust
Contact the on-call Ophthalmology and/or Maxillofacial surgery (MaxFax) team (available 24/7)
Early referral is essential

## Slide 12
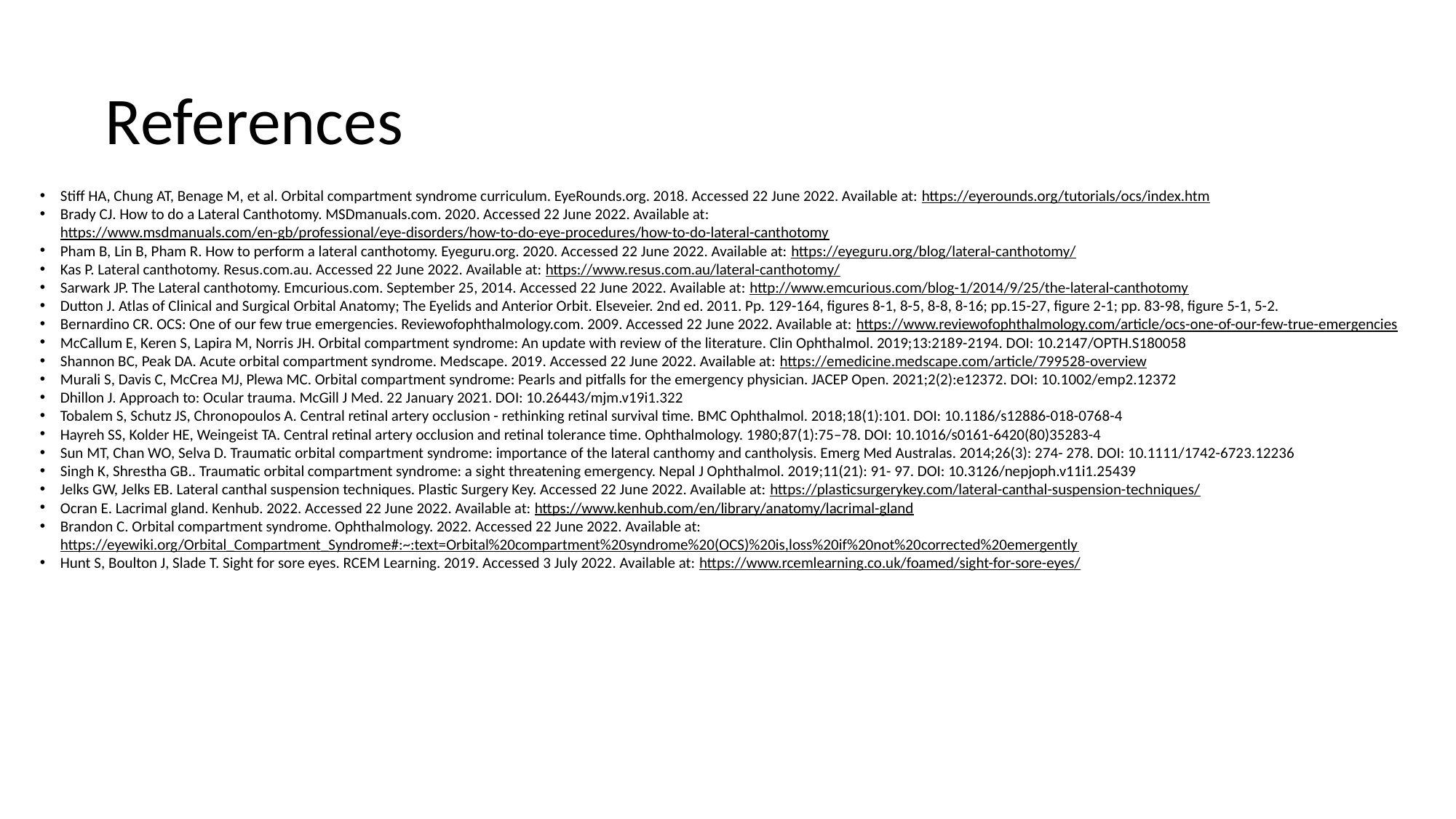

# References
Stiff HA, Chung AT, Benage M, et al. Orbital compartment syndrome curriculum. EyeRounds.org. 2018. Accessed 22 June 2022. Available at: https://eyerounds.org/tutorials/ocs/index.htm
Brady CJ. How to do a Lateral Canthotomy. MSDmanuals.com. 2020. Accessed 22 June 2022. Available at: https://www.msdmanuals.com/en-gb/professional/eye-disorders/how-to-do-eye-procedures/how-to-do-lateral-canthotomy
Pham B, Lin B, Pham R. How to perform a lateral canthotomy. Eyeguru.org. 2020. Accessed 22 June 2022. Available at: https://eyeguru.org/blog/lateral-canthotomy/
Kas P. Lateral canthotomy. Resus.com.au. Accessed 22 June 2022. Available at: https://www.resus.com.au/lateral-canthotomy/
Sarwark JP. The Lateral canthotomy. Emcurious.com. September 25, 2014. Accessed 22 June 2022. Available at: http://www.emcurious.com/blog-1/2014/9/25/the-lateral-canthotomy
Dutton J. Atlas of Clinical and Surgical Orbital Anatomy; The Eyelids and Anterior Orbit. Elseveier. 2nd ed. 2011. Pp. 129-164, figures 8-1, 8-5, 8-8, 8-16; pp.15-27, figure 2-1; pp. 83-98, figure 5-1, 5-2.
Bernardino CR. OCS: One of our few true emergencies. Reviewofophthalmology.com. 2009. Accessed 22 June 2022. Available at: https://www.reviewofophthalmology.com/article/ocs-one-of-our-few-true-emergencies
McCallum E, Keren S, Lapira M, Norris JH. Orbital compartment syndrome: An update with review of the literature. Clin Ophthalmol. 2019;13:2189-2194. DOI: 10.2147/OPTH.S180058
Shannon BC, Peak DA. Acute orbital compartment syndrome. Medscape. 2019. Accessed 22 June 2022. Available at: https://emedicine.medscape.com/article/799528-overview
Murali S, Davis C, McCrea MJ, Plewa MC. Orbital compartment syndrome: Pearls and pitfalls for the emergency physician. JACEP Open. 2021;2(2):e12372. DOI: 10.1002/emp2.12372
Dhillon J. Approach to: Ocular trauma. McGill J Med. 22 January 2021. DOI: 10.26443/mjm.v19i1.322
Tobalem S, Schutz JS, Chronopoulos A. Central retinal artery occlusion - rethinking retinal survival time. BMC Ophthalmol. 2018;18(1):101. DOI: 10.1186/s12886-018-0768-4
Hayreh SS, Kolder HE, Weingeist TA. Central retinal artery occlusion and retinal tolerance time. Ophthalmology. 1980;87(1):75–78. DOI: 10.1016/s0161-6420(80)35283-4
Sun MT, Chan WO, Selva D. Traumatic orbital compartment syndrome: importance of the lateral canthomy and cantholysis. Emerg Med Australas. 2014;26(3): 274- 278. DOI: 10.1111/1742-6723.12236
Singh K, Shrestha GB.. Traumatic orbital compartment syndrome: a sight threatening emergency. Nepal J Ophthalmol. 2019;11(21): 91- 97. DOI: 10.3126/nepjoph.v11i1.25439
Jelks GW, Jelks EB. Lateral canthal suspension techniques. Plastic Surgery Key. Accessed 22 June 2022. Available at: https://plasticsurgerykey.com/lateral-canthal-suspension-techniques/
Ocran E. Lacrimal gland. Kenhub. 2022. Accessed 22 June 2022. Available at: https://www.kenhub.com/en/library/anatomy/lacrimal-gland
Brandon C. Orbital compartment syndrome. Ophthalmology. 2022. Accessed 22 June 2022. Available at: https://eyewiki.org/Orbital_Compartment_Syndrome#:~:text=Orbital%20compartment%20syndrome%20(OCS)%20is,loss%20if%20not%20corrected%20emergently
Hunt S, Boulton J, Slade T. Sight for sore eyes. RCEM Learning. 2019. Accessed 3 July 2022. Available at: https://www.rcemlearning.co.uk/foamed/sight-for-sore-eyes/
